# Transcriptional and genetic alterations of cuproptosis-related genes correlated to malignancy and immune-infiltrate of esophageal carcinoma

**DOI:** 10.1038/s41420-022-01164-5

**Published:** 2022-08-22

**Authors:** Runmin Jiang, Yu Huan, Yan Li, Xinyue Gao, Qiang Sun, Feng Zhang, Tao Jiang

**Affiliations:** 1 Department of Thoracic Surgery, Tangdu Hospital, The Fourth Military Medical University, Xi’an, China; 2 State Key Laboratory of Cancer Biology, Department of Pathology, Xijing Hospital and School of Basic Medicine, The Fourth Military Medical University, Xi’an, Shaanxi China; 3 Department of Neurosurgery, Xijing Hospital, The Fourth Military Medical University, Xi’an, China; 4 Department of Radiation Oncology, Xijing Hospital, The Fourth Military Medical University, Xi’an, China; 5 Laboratory of Cell Engineering, Institute of Biotechnology; Research Unit of Cell Death Mechanism, 2021RU008, Chinese Academy of Medical Science, Beijing, China

**Keywords:** Oesophageal cancer, Tumour immunology

## Abstract

Esophageal carcinoma (ESCA) is a common type of cancer with high mortality. Cuproptosis is a new type of cell death and is characterized by the dependence on mitochondrial respiration and protein lipoylation. However, the potential roles of cuproptosis-related genes (CRGs) in ESCA remain elusive. Here, we systematically assessed the transcriptional and genetic alterations of CRGs in ESCA. We identified a CRGs signature for ESCA patients. A 6-CRGs signature was constructed by the least absolute shrinkage and selection operator (LASSO) regression analysis along with the univariate cox regression analysis and differential genes analysis. The CRGs score could significantly stratify ESCA patients’ survival and a high CRGs score was significantly correlated with worse overall survival. Moreover, higher CRGs score indicated higher pathology grades and aberrant cell adhesion, possibly via the PI3K-AKT pathway, which could also underly their increased sensitivity to PI3K-AKT pathway inhibitors. In addition, patients with high CRGs tend to hold more mutation load and abnormal APOBEC mutation. Notably, a higher CRGs score was anomalously associated with more immune infiltration, which could explain its malignancy by increased PD-L1 stability and a higher proportion of bystander T cells. In conclusion, our report revealed the significance of cuproptosis in ESCA and may have therapeutic potential in activating the bystander T cells.

## Introduction

Esophageal carcinoma (ESCA) is one of the most common cancers worldwide, ranking seventh in morbidity and sixth in mortality among all cancers [1, 2]. Due to the lack of early diagnosis, most patients (~75%) are diagnosed at late stages, which resulted in only about 20–25% five-year survival rate for decades without great improvement [3]. Thus, a more useful prognostic biomarker, and therapeutic strategy as well, is still a pressing need.

Copper is one of the most important metals in bioactivities. Cells have to maintain a modest copper concentration for survival and function normally. It would result in life-threatening diseases once the copper homeostasis was dysregulated such as genetic variation [4, 5]. The function of key metal-binding enzymes will be damaged in a low copper concentration. On the contrary, the threshold-exceeded accumulation of copper would also be toxic and lead to cell death [6]. Cuproptosis is a new type of cell death and is characterized by the dependence on mitochondrial respiration and protein lipoylation, which is distinct from known death mechanisms, including apoptosis, ferroptosis, pyroptosis, necroptosis, and non-cell-autonomous death mediated by cell-in-cell structures [6–12]. Once overloaded, copper would bind directly to lipoylated components of the tricarboxylic acid (TCA) cycle, which leads to lipoylated protein aggregation and subsequent loss of iron-sulfur cluster protein. This would contribute to proteotoxic stress, ultimately resulting in cell death [6].

Accumulating evidence indicates the crosstalk between cuproptosis and cancer. Elesclomol, a molecule that binds copper in the environment and brings it into the cell to induce cell death, has been employed in human clinical trials for epithelial cancer therapy [13]. According to Tsvetkov’s reports, elesclomol was supposed to work best in cancers with up-regulation of lipoylated mitochondrial proteins and highly respiratory, which was supposed to be especially useful for cancers that are naturally resistant to apoptosis [6]. However, the applications of cuproptosis in ESCA were yet to be explored.

Here, by comprehensive analysis of the cuproptosis-related genes (CRGs) in ESCA, we reported that the higher GSVA score of the cuproptosis pathway indicates the progression of ESCA cells such as higher pathology grades and aberrant cell adhesion, possibly via the PI3K-AKT pathway. We showed a positive correlation between PI3K-AKT pathway inhibitors sensitivity and CRGs expression in ESCA cells. Additionally, we developed a CRGs signature as a promising prognostic model by the least absolute shrinkage and selection operator (LASSO). The higher CRGs score suggested a bad survival ending while a more immune infiltration in ESCA, which could be explained by the increased stability of PD-L1 and the increased proportion of bystander T cells. Our analysis revealed the significance of cuproptosis in ESCA and may have therapeutic potential in activating the bystander T cells in ESCA.

## Results

### The transcriptional and genetic landscape of cuproptosis-related genes (CRGs) in ESCA

**Fig. 1:**
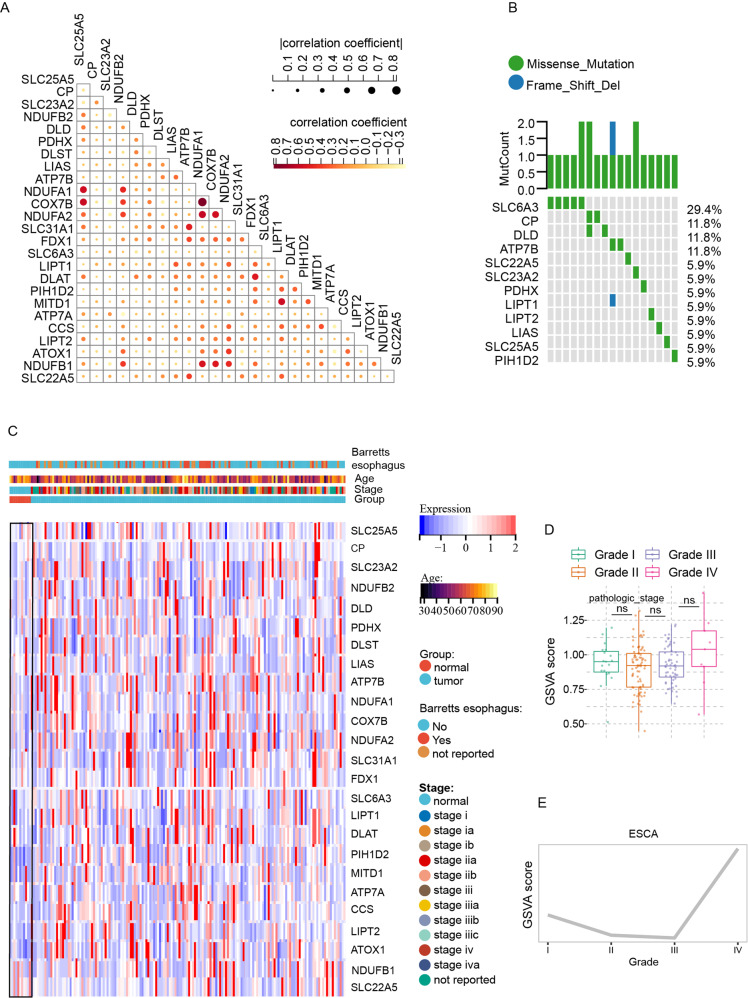
The transcriptional and genetic alterations of cuproptosis-related genes in esophagus carcinoma. **A** The correlations between the expression of cuproptosis-related genes (CRGs). **B** Mutation frequencies of 25 CRGs in 173 patients with esophagus carcinoma (ESCA). **C** The heatmap showed the expression level and clinicopathologic features of 25 cuproptosis-related genes between normal and ESCA samples. **D**, **E** Differences (**D**) and tendency (**E**) in GSVA score of 25 CRGs in ESCA samples with different pathology grades. Data was exhibited as mean ± SD. Significance was assessed by Pearson’s correlation test (**A**), and KruskalWallis test (**D**). ns = not significant, *P < 0.05; **P < 0.01; ***P < 0.001.

Firstly, we collected a total of 25 CRGs for analysis based on the cuproptosis pathway and Genecard database (Table S1). In order to further explore the expression correlations of CRGs, we conducted a correlation analysis and showed the results in Fig. 1A, respectively. To investigate the global expression of CRGs, we compared the expression level of CRGs between the normal and ESCA samples. Heatmap revealed an increased overall expression level of CRGs in ESCA samples (Fig. 1C). In line with this, although there was no significance of GSVA scores among the different grades, it still revealed an increased tendency of GSVA scores in the higher grades in ESCA (Fig. 1D, E).

### Identification of candidate prognostic CRGs

**Fig. 2:**
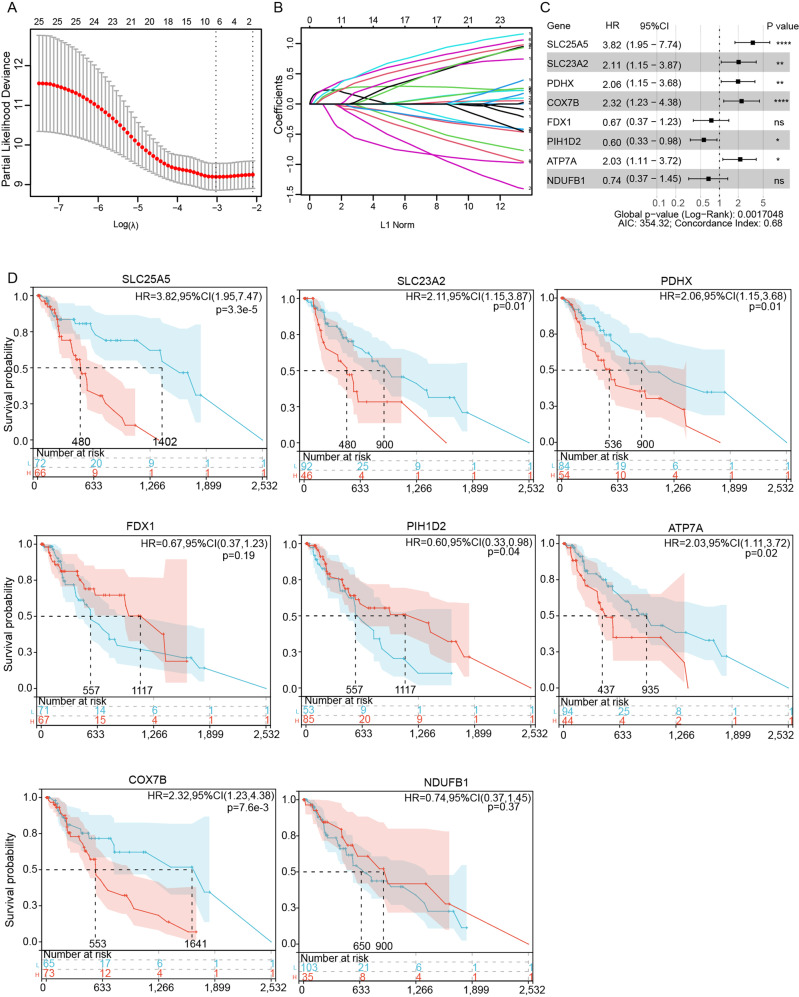
Identification of candidate prognostic CRGs. **A**, **B** LASSO analysis filtered out the candidate prognostic CRGs of the 25 CRGs. **C** Univariate Cox analysis of the candidate prognostic CRGs. **D** The Kaplan-Meier curves of the candidate prognostic CRGs for ESCA patients. Significance was assessed by a log-rank test (**B**).

Next, we explored the relationship between the CRGs expression and patients’ survival. From the 25 CRGs, eight genes SLC25A5, SLC23A2, PDHX, COX7B, PIH1D2, FDX1, ATP7A, NDUFB1 (*SLC25A5, SLC23A2, PDHX, COX7B, PIH1D2, FDX1, ATP7A*, and *NDUFB1*) were ultimately filtered to be associated with prognosis using LASSO regression analysis (Fig. 2A, B, Table S2). Meanwhile, univariate Cox regression analysis and Kaplan–Meier survival curves were further utilized to assess the significance of the eight CRGs expression on the prognosis of ESCA patients. As shown in Fig. 2C, D, the higher expression of *SLC25A5, SLC23A2, PDHX*, ATP7A, and *COX7B* predicted a bad survival ending (*P* < 0.05) while patients would obtain a longer survival when the expression of *PHID2* was higher (*P* < 0.05).

### Construction and validation of a minimal CRGs signature that predicted the survival of ECSA patients

**Fig. 3:**
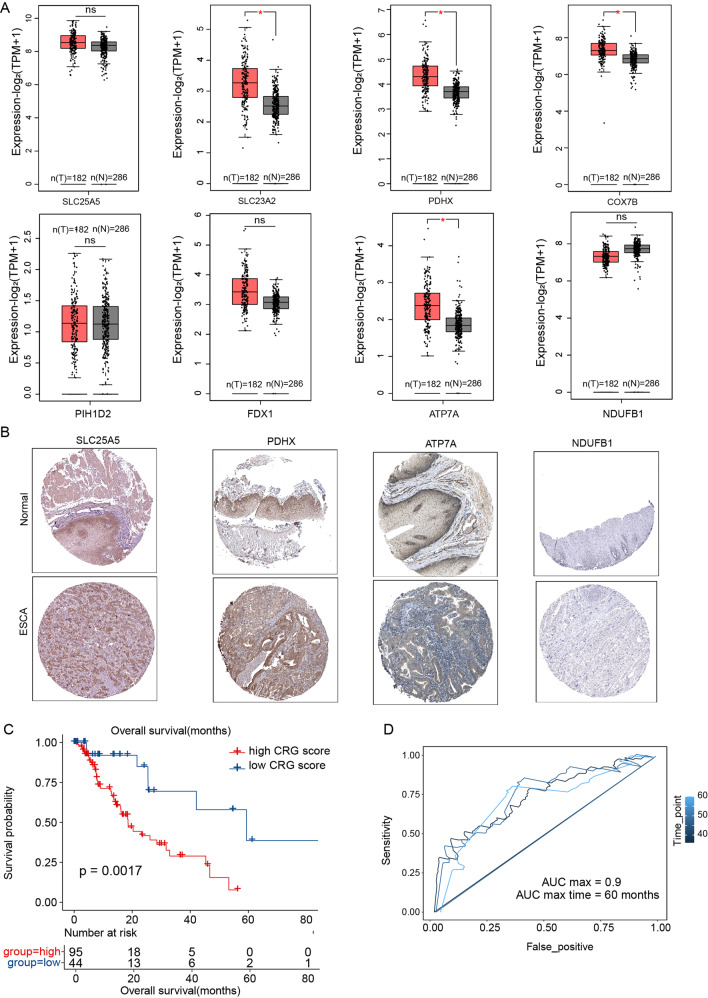
Construction of the minimal CRGs signature that predicted the survival of ECSA patients. **A** Differential gene expression of the prognostic CRGs between normal samples and ESCA samples. The data was from the GEPIA2 database. **B** Representative images showed the differential protein level of the prognostic CRGs between normal samples and ESCA samples. The data was from The Human Protein Atlas database. **C** Kaplan-Meier survival curves of the CRGs- signature in ESCA patients. **D** The 3-, 4-, and 5-year ROC curves for CRGs- signature in ESCA patients. Data was exhibited as mean ± SD. Significance were assessed by Wilcoxon test (**A**) and log-rank test (**C**). ns = not significant, *P < 0.05; **P < 0.01; ***P < 0.001.

In order to further confirm the expression levels of the eight genes, we visualized their mRNA expression levels and found the expression of *SLC23A2, PDHX*, *COX7B*, and *ATP7A* up-regulated in tumor, while the expression of the other showed no significant difference (Fig. 3A). Consistently, the representative immunohistochemical staining images of these genes from The Human Protein Atlas also showed a similar expression trend (Fig. 3B). We, therefore, selected the six genes (*SLC25A5, SLC23A2, PDHX, COX7B, ATP7A, PIH1D2*) as the minimal CRGs and the corresponding risk score formula was as follows: Risk score = 0.22863 × expression of *SLC25A5* + 0.18401 ×expression of *SLC23A2* + 0.33753× expression of *PDHX* + 0.28295 × expression of *COX7B* + 0.23044 ×expression of *ATP7A* -0.02041 × expression of *PIH1D2*. 95 patients were assigned to the high CRGs-score group, and the other 44 patients were assigned to the low CRGs -score (Fig. 3C). Notably, the 6 CRGs signature could significantly stratify ESCA patients into two prognostic groups with high scores predicting shorter overall survival (Fig. 3C). Consistently, the 3-, 4-, and 5-year ROC curves demonstrated that the CRGs-signature held a good prediction performance (*p* < 0.05, AUC_max_ = 0.9, Fig. 3D)

### Association of high CRGs score with abnormal cell adhesion and PI3K-AKT pathway

**Fig. 4:**
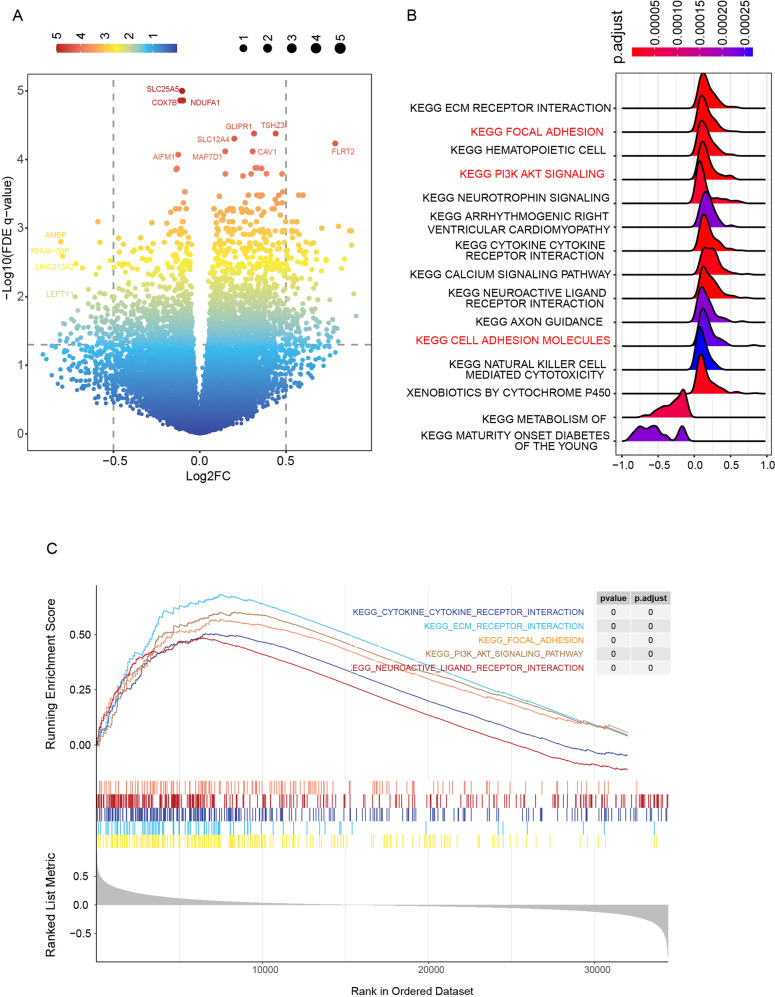
Higher CRGs score was related to abnormal cell adhesion via the Akt-PI3K pathway. **A** Differentially expressed genes (DEGs) between the high-CRGs score group and the low-CRGs score group. **B** KEGG enrichment analyses of DEGs between high-CRGs score group and low-CRGs score group. **C** GSEA enrichment analyses of DEGs between high-CRGs score group and low-CRGs score group.

To investigate the genes and pathways that underlie the survival difference, we firstly identified the differential genes between the high and low CRGs-score groups. Except for CRGs (*SLC25A5, COX7B*), *AMBP, AIFM1, MAP7D1, TSHZ3, CAV1*, et al. showed a significant difference (Fig. 4A). Further KEGG along with the GSEA analysis revealed that the differential genes between high and low CRGs group were mainly involved in the regulation of ECM receptor, focal adhesion, cell adhesion, and PI3K-AKT signaling pathway (Fig. 4B). Thus, we suppose that the different survival endings of two CRGs-score groups may ascribe to the aberrant function in cell adhesion, which possibly results from the activation of PI3K-AKT signaling.

### Association of high expression of CRGs with TGX221 sensitivity

**Fig. 5:**
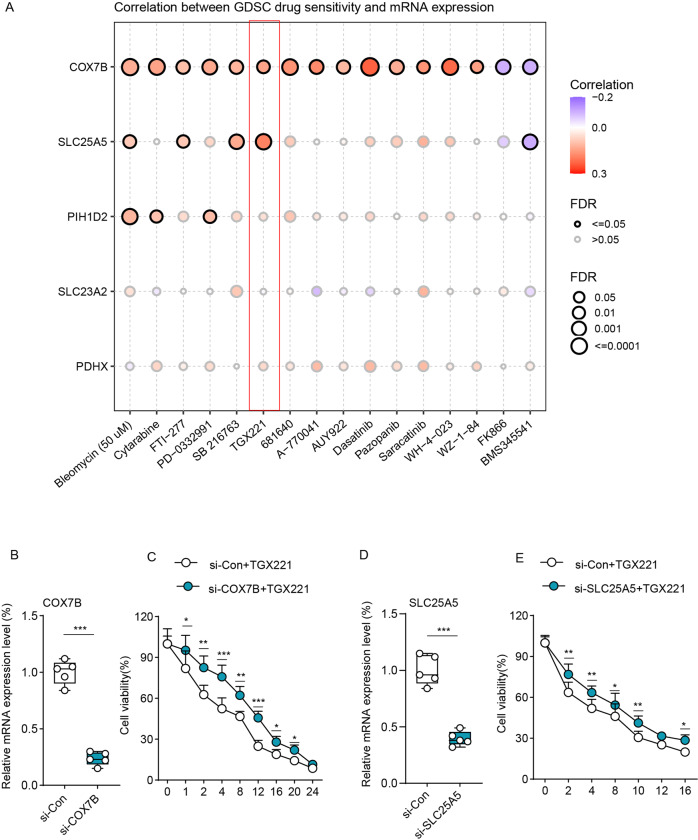
Higher expression of CRGs was related to more drug sensitivity. **A** The correlations between the drug sensitivity and CRGs expression. **B**, **C** qRT-PCR analysis verified the efficiency of COX7B (**B**) and SLC25A5 (**C**) knockdown in EC109 cells. **D** Cell viability between EC109 cells transfected with si-Con1 and si-COX7B when treated with a gradient dose of TGX221 (*n* = 5). **E** Cell viability between EC109 cells transfected with si-Con2 and si-SLC25A5 when treated with a gradient dose of TGX221 (*n* = 5). Data was exhibited as mean ± SD. Significance were assessed by two-tailed *t*-test (**D**, **E**). ns = not significant, *P < 0.05; **P < 0.01; ***P < 0.001.

To investigate the potential drugs for ESCA therapy, we used the Genomics of Drug Sensitivity in Cancer (GDSC) database to assess the relationship between CRGs and drug sensitivity. As shown in Fig. 5A, higher expression of *COX7B* was correlated with higher sensitivity of various drugs, such as Bleomycin, TGX221, and Dasatinib. Similarly, there are also positive correlations between the *SLC25A5* or *PIH1D2* expression and the drug sensitivity. Consistent with the Fig. 4B, among these drugs, TGX221 and SB 216763 were the PI3K-AKT pathway inhibitors, which showed a great positive correlation with COX7B or SLC25A5 expression. Thus, we selected the TGX221 for further experiments. To validate the positive correlations between TGX221 and COX7B, we transfected si-COX7B into EC109 cells and then verify the efficiency by qRT-PCR (Fig. 5B). Next, we treated si-Con1 EC109 cells and si-COX7B EC109 cells with a gradient dose of TGX221 and found that si-COX7B EC109 cells showed more cell viability when treated with TGX221, indicating its lower sensitivity (Fig. 5C). Similar results were also observed in si-SLC25A5 EC109 cells (Fig. 5D, E).

### A high mutation load in patients with high CRGs score

**Fig. 6:**
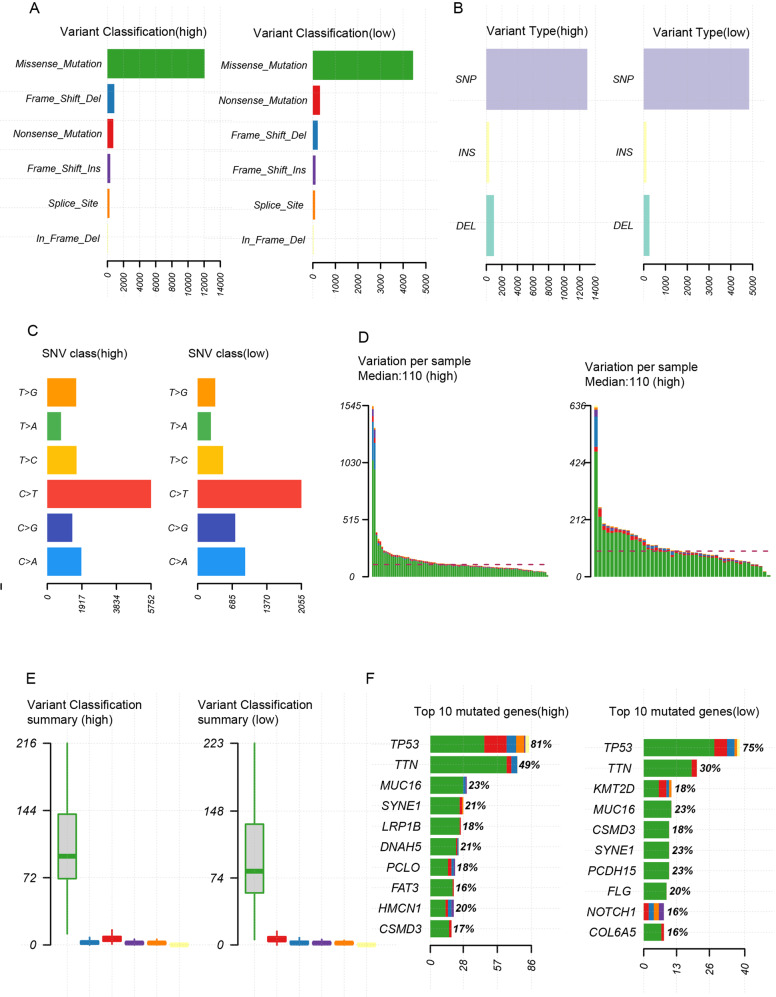
Higher CRGs score patients held a higher mutation loading. **A** Variant classification graph between high-CRGs score group and low-CRGs score group. **B** Variant type graph between high-CRGs score group and low-CRGs score group. **C** SNV class graph between high-CRGs score group and low-CRGs score group. **D** Variation per sample between high-CRGs score group and low-CRGs score group. **E** Variant classification summary graph between high-CRGs score group and low-CRGs score group. **F** Top 10 mutated genes between high-CRGs score group and low-CRGs score group.

We then explore the genetic alterations in the two CRGs-score groups by taking advantage of the TCGA database. As shown in Fig. 6A–D, patients with high CRGs score obtained a higher loading of various variant classifications and types (missense mutation, frameshift deletions, nonsense mutation, SNP, INS, DEL). The top ten mutated genes in the high group are *TP53, TTN, MUC16, SYNE1, LRP1B, DNAH5, PCLO, FAT3, HMCN1, CSMD3*, and the low group has *TP53, TTN, KMT2D, CSMD3, MUC16, SYNE1, PCDH15, FLG, NOTCH1, COL6A5* (Fig. 5E). Patients with high CRGs score had markedly higher frequencies of *TP53(81%), and TTN (49%)* mutations compared to *TP53(75%), and TTN (30%)* in the low CRGs-score group. Moreover, *LRP1B, DNAH5, PCLO, FAT3, HMCN1* mutations were seems to be peculiar to high CRGs core patients, indicating their association with the CRGs and worse bio-activities in ESCA (Fig. 6E).

### APOBEC family mutation landscape in two CRGs-score groups

**Fig. 7:**
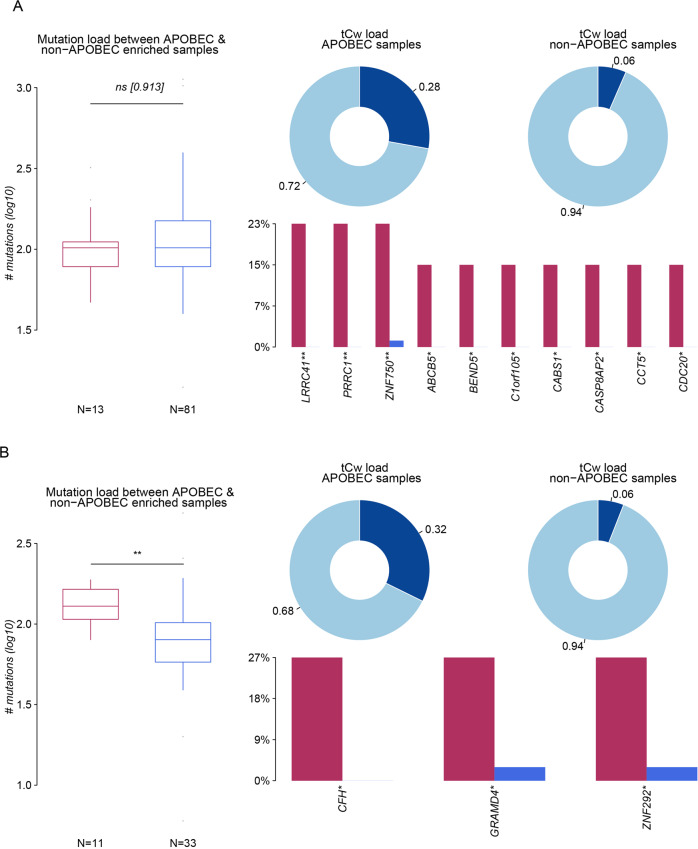
APOBEC family mutation landscape in two CRGs -score groups. **A** The mutation load and tCw load between the APOBEC and non-APOBEC enriched samples in the high-CRGs score group. **B** The mutation load and tCw load between the APOBEC and non-APOBEC enriched samples in the low-CRGs score group. Data was exhibited as mean ± SD. Significance were assessed by Wilcoxon test (**A**, **B**). ns = not significant, *P < 0.05; **P < 0.01; ***P < 0.001.

APOBEC (apolipoprotein B mRNA-editing enzyme catalytic polypeptide) is a family of cytidine deaminase, whose transcription can be activated by pro-inflammatory cytokines and chemokines [14]. It was found that the APOBECs family could drive the formation of cancer-promoting virus mutants. Its gene coding function could also induce cancer-promoting driving mutation which plays a bridge role in inflammation-cancer transformation [15, 16]. In the high CRGs score group, the mutation load between APOBEC-enriched and non-enriched samples showed no significance and the TCW loading rate (C > T and C > G mutations at T-C-A/T trinucleotides) in APOBEC-enriched samples was 0.28, which was lower than that in low CRGs score group (0.32). However, many mutated APOBEC genes in the high CRGs score group (no less than 10) were higher than the only 3 mutated APBEOC genes in the low CRGs group, which may also underly the tumor progression in the high CRGs score group (Fig. 7A, B).

### Association of high CRGs score with immune infiltration, PD-L1 stability, and an increased proportion of bystander T cells

**Fig. 8:**
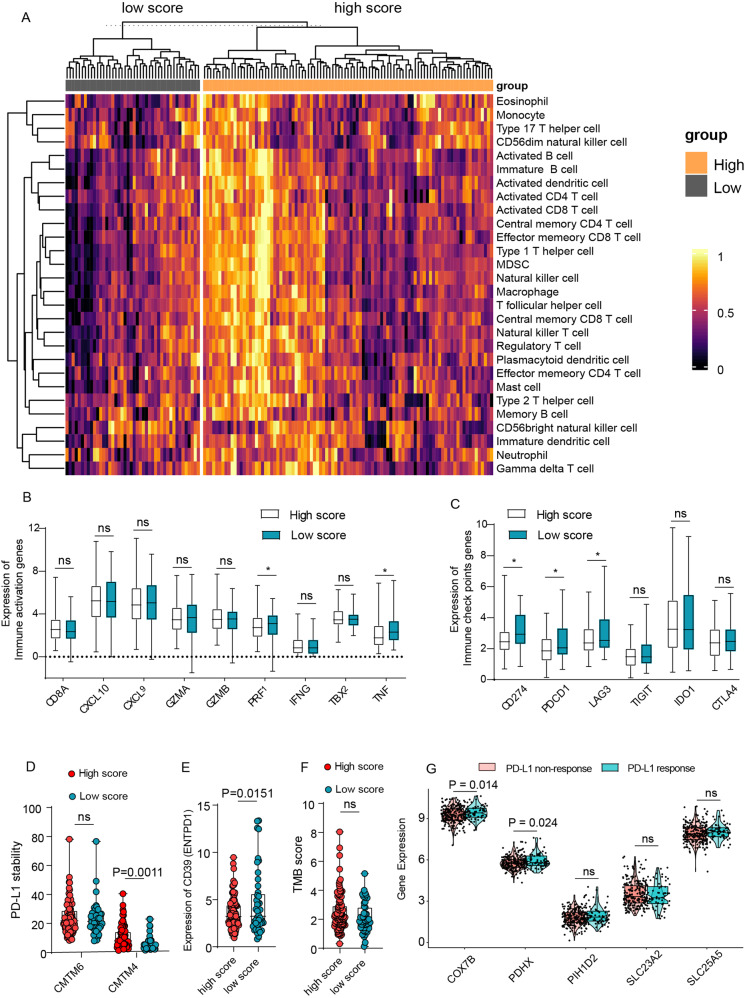
Higher CRGs score was associated with more immune infiltration, PD-L1 stability, and more proportion of bystander T cells. **A** The heatmap showed the immune infiltration status between the high-CRGs score group and the low-CRGs score group. **B** Expression of immune activation genes between high-CRGs score group and low-CRGs score group. **C** Expression of immune checkpoint genes between high-CRGs score group and low-CRGs score group. **D** Expression of PD-L1 stability genes (CMTM6, CMTM4) between high-CRGs score group and low-CRGs score group. **E** Expression of bystander T cell genes (CD39) between high-CRGs score group and low-CRGs score group. **F** Tumor mutation burden (TMB) score between high-CRGs score group and low-CRGs score group. **G** Expression of CRGs between response and non-response patients that received PD-L1 inhibitor treatment in the IMvigor210 (mUC) cohort. Data was exhibited as mean ± SD. Significance were assessed by two-tailed *t*-test (**B**–**G**). ns = not significant, *P < 0.05; **P < 0.01; ***P < 0.001.

To investigate the immune infiltration status between high and low CRGs score groups, we next performed ssGSEA analysis of immune cells-related genes to assess the association between CRGs score and the infiltration of immune cells. Unexpectedly, as shown in Fig. 8A, the heat map of immune cells exhibited a much more infiltration in the high CRGs-score group, such as activated CD4 + T cells, activated CD8 + T cells, memory T cells, and NK cells. Given that the high- CRGs score implies a bad survival ending, it seems that more infiltration could not explain it well. To solve this problem, we investigated the immune activation status by comparing the expression of CD8A, CXCL9, CXCL10, GZMA, GZMB, PRF1, and IFHG, TBX2, and TNF. As expected, the immune activation status showed no difference between the high and low CRGs score group (Fig. 8B). In addition, although the immune checkpoints genes such as PD-L1 expression were downregulated in the higher CRGs group, the stability of PD-L1 was upregulated in these samples due to the upregulation of CMTM6 and CMTM4, which could prevent PD-L1 from being targeted for lysosome-mediated degradation [17] (Fig. 8C, D). Furthermore, the down-regulation of CD39 also suggests a higher proportion of bystander T cells, which may underly this problem as well (Fig. 8E). Next, we checked the tumor mutation burden (TMB) score to evaluate the PD-L1 effect on ESCA. Although there was no difference between the high and low CRGs score group, the high CRGs-score group appeared to hold a higher TMB score (Fig. 8F). Consistently, it was also found in the ICVRG2010 cohort that the CRGs expression (COX7B, PDHX) in PD-L1 response patients was higher than in non-response patients, indicating a preferable curative potency and better survival ending for patients with higher CRGs score when treated with PD-L1 (Fig. 8G). Taken together, these results revealed that a high CRGs score was associated with higher immune infiltration and bystander T cells, and patients may be more responsive to anti-PD-L1 therapy.

## Discussion

Previous studies suggested aberrant copper homeostasis (ACH) was highly associated with cancers such as bladder cancer, breast cancer, colorectal cancer, and prostate cancers [18–21]. Supporting this, in carcinogenesis, ACH was observed to contribute to the proangiogenic response via various molecular pathways, which played a fundamental role in cancer proliferation or angiogenesis [22]. Moreover, copper accumulation in cancer cells was also reported [23, 24].

Here, we systematically investigate the role of the cuproptosis-related genes in the prognosis, pathways, and immune infiltration of ESCA. By performing LASSO analysis along with exploration of their differential expression in mRNA and protein level, we identified a 5 CRGs signature. The KM curve suggested that the CRGs signature could stratify overall survival efficiently.

*SLC25A5, SLC23A2, PDHX, COX7B, ATP7A*, and *PIH1D2* were filtered out to construct the CRGs signature, which was rather reasonable. *SLC25A5* was identified as a biomarker in clear cell renal cell carcinoma involving competitive endogenous RNA [25]. *SLC23A2(SVCT2)*, which takes the function to reduce the oxidative damage caused by copper overloaded [26, 27], was reported to be a polymorphism in gastric cancer or chronic lymphocytic leukemia [28, 29]. *PDHX*, which takes part in encoding the pyruvate dehydrogenase (PDH) complex, was deficient in human breast tumor samples, and low levels of PDHX were also associated with decreased patient survival [30]. Consistently, in nasopharyngeal carcinoma (NPC), COX7B was found to hold a high positive rate (84.24%) in tumor samples and was thought to be a putative molecular marker in NPC [31]. *PIH1D2* was reported to be deleted in paragangliomas [32].

Our reports revealed that CRGs was also associated with the grading of ESCA. On one hand, the high grades samples of ESCA appeared to hold a higher GSVA score of CRGs (Fig. 1 C, D). On the other, the differential genes between high and low CRGs-score groups showed enrichment in extracellular matrix interaction and focal adhesion function, suggesting an aberrant cell adhesin in high CRGs score samples. Consistent with our reports, Cu concentration was also found to increase in tumor areas and even correlated with the grade of cancer [33–35]. High serum Cu levels were also found in cancer patients resistant to chemotherapy compared to patients responding to treatment [35] and promote the scatter and formation of secondary tumors by activating cell proliferation-related enzymes [22].

Interestingly, in our analysis, patients with high CRGs score gained more immune infiltration while a bad survival ending. Although the immune-escaping genes such as PD-L1(*CD274*), and *LAG3* in high CRGs score patients were downregulated, the PD-L1 would be more stable in these patients due to the high expression of *CMTM4*, which is regarded as the PD-L1 stabler. Consistent with our reports. Zhou B also found that Cu ionophore disulfiram can induce stabilization of PD-L1 by overloading cancer cells with Cu [36]. Moreover, bystander T cells were often observed in cancers, such as lung cancer and colon cancer, which represented a poor immunotherapy response [37, 38]. Here, we found the CRGs score could filter patients with bystander T cells, due to the high CRG score patients held the lower expression of CD39 (*ESPDN1*), the marker of bystander T cells [37, 38]. Nonetheless, the relationship between cuproptosis and the bystander T cells should be further explored.

In conclusion, our study comprehensively explored the expression profiling and mutation landscape of cuproptosis-related genes in ESCA. By constructing the cuproptosis-related genes (CRGs) signature, we reported that a higher CRGs score represented the progression of ESCA, because worse survival outcomes, higher pathology grades, aberrant cell adhesion and APOBEC mutation loading were found in patients with high CRGs scores. In addition, higher CRGs score patients tend to hold more immune infiltration, which we analyzed that the higher proportion of bystander T cells and the more stable PD-L1 expression would underlie it. Our results provide a new prognostic predictor and offer novel insights into the clinical application of cuproptosis and immune checkpoints targeting therapies.

## Materials and methods

### Data source

The CRGS were collected from Tsvetkov’s reports [6] and Genecard. A total of 25 CRGs were included in this study (Table S1). The gene expression data and clinical data were downloaded from The Cancer Genome Atlas database (TCGA) up to March 1, 2022 (https://portal.gdc.cancer.gov). A total of 174 samples were collected, including 11 normal samples and 163 tumor samples. Transcripts per million (TPM) of gene expression data was utilized for further analysis. The clinical data includes the age, clinical stages, pathology grades, and Barrett’s esophagus.

GEPIA2 was used to compare the expression levels of genes between normal and tumor samples. Human Protein Atlas (HPA) was utilized to compare the expression level of the protein between normal and tumor samples.

The mutation annotation format (MAF) data of ESCA was downloaded from the UCSC Xena server and analyzed with the “maftools” R package. Oncoplot was drawn according to the descending order of mutations.

To compare the gene expression between response and non-response patients treated with a PD-L1 inhibitor, the data of the IMvigor210 (mUC) cohort from patients with mUC receiving PD-L1 inhibitor treatment were collected. The expression data and clinical data were obtained from the IMvigor210 (mUC) dataset (http://research-pub.gene.com/IMvigor210CoreBiologies) with the ‘IMvigor’ package in R.

### Construction of the CRGs signature

To reduce the risk of over-fitting, the least absolute shrinkage and selection operator (LASSO) with 10-fold cross-validation was performed with the “glmnet” R package screening for CRGs related to overall survival as described [39]. Next, univariate Cox regression analysis were conducted to assess whether this risk score model displayed good predictive ability for prognosis. Then, CRGs with no significance in their expression between normal and ESCA tumors were excluded. Next, The CRGs-score of each patient was calculated based on the amount of calculated gene expression and the corresponding coefficient. The formula was as the following:

$$CRG\_score = {\sum} {\left( {{{{\mathrm{Expi}}}} \ast {{{\mathrm{coefi}}}}} \right)}$$

where Expi and Coefi represented the expression of each gene and the risk coefficient, respectively. Next, we classified the patients into high-risk group and low-risk group according to the optimal cutoff value of risk score that was analyzed by the R package “survival”. Kaplan–Meier analysis was carried out to explore the prognostic significance of the CRGs signature in ESCA by the “ggsurvplot” package. Then, to assess the predictive efficiency of the CRGs-signature, receiver operating characteristic (ROC) of 5-year survival was performed with the “survivalROC” package in the TCGA-ESCA cohort.

### Differential genes exploration and gene set enrichment analysis

Differential genes between the high CRGs score and low CRGs score group were identified by the “limma” package in R with the criterion of |log2(fold change) | > 0.5 and adjusted *p* value < 0.05.

Furthermore, we performed the Kyoto Encyclopedia of Genes and Genomes (KEGG) pathway enrichment analysis and gene set enrichment analysis (GSEA) of CRGs with the R package “clusterProfiler”. *P* < 0.05 and the false discovery rate (FDR q) < 0.05 was considered statistically significant.

### Evaluation of chemotherapeutic drug sensitivity

Genomics of Drug Sensitivity in Cancer (GDSC) (https://www.cancerrxgene.org/) were used to explore the relationship between mRNA expression level and drug sensitivity. To be specific, the IC50 of small molecules in various cell lines and the mRNA gene expression were obtained from GDSC. Then, Pearson analysis was carried out to evaluate the correlation between gene expression and IC50. *P*-value was adjusted by FDR.

### Cell culture

The human esophagus carcinoma cell line (ECA-109) was obtained from the American Type Culture Collection (ATCC). The identity of the EC109 cell line was validated to be correct by Short tandem repeat (STR). Cells were cultured with DMEM (Gibco, ThermoFisher Scientific, Waltham, USA) medium supplemented with 10% fetal bovine serum, 50 μg ml^−1^ streptomycin, 100 units ml-1 penicillin, (Gibco), and incubated at 37 °C with 5% CO_2_.

### Cell transfection

siRNA of candidate genes and the control si-Con were commercially purchased from Tsingke (Tsingke Biotechnology Co., Ltd., Beijing, China). Cells were transfected according to the protocols of the ViaFect™ (Promega, Wisconsin, USA). The sequences of siRNA were shown in Table S2

### RNA isolation and quantitative real-time PCR

Total cellular RNA was extracted using the TRIzol reagent (Invitrogen, Carlsbad, CA, USA). The Complementary DNA (cDNA) was synthesized with the PrimeScript RT reagent kit (Takara, 6210, China). The RT-qPCR was performed with SYBR Green Supermix (Bio-Rad, 172-5850, USA). The gene expression levels data were calculated using the 2-ΔΔC t method, normalized with GAPDH expression levels. The primer sequences used are listed in Table S3.

### Drug sensitivity measurements

Drug sensitivity was determined using the cell counting kit-8 assay (CCK-8 assay kit, Bimake, Houston, USA). Cells were seeded in 96-well plates at a density of 5000 cells per well and cultured overnight for adhesion. Then, gradient doses of TGX221(Selleck, USA, Texas) were added to the culture medium for 24 h. Next, 2 h after 10 µl of CCK-8 administration per well, the optical density was measured at 450 nm with a microplate reader with a spectrophotometer (Mutiskan Go, ThermoFisher).

### Immune infiltration analysis

The set of marker genes for 28 immune-related cells and types were collected from Jia et al. [40]. The ssGSEA method of the Gene Set Variation Analysis (GSVA) package was applied to analyze the infiltration level of different immune cells in high CRG-score and low-CRGs score expression profile data.

### Statistical analysis

Data was exhibited as mean ± SD. Pearson’s correlation test was used to assess the correlation between CRGs expression levels in ESCA samples. Levene test was used to assess the variance homogeneity of data in different groups. Student t tests or Wilcoxon tests were utilized to estimate the significance between the two groups. In addition, Kruskal-Wallis’s test was used for comparing more than two variables in this study (Fig. 1D). Significance between Kaplan-Meier survival curves was determined with the log-rank test. Univariate Cox regression analysis was used to estimate the hazard ratio (HR) and 95% confidence interval (95% CI). Mann-Whitney U test was employed to compare the number of somatic mutations. All statistical analysis were performed with R (v 4.1.0). *p* < 0.05 is considered to be a statistically significant.

## Supplementary information

Supplemental Table Legends

Gene symbol list

The sequence of siRNA

Primers for PCR

## Data Availability

The datasets in this study are publicly available. They can be found at the location described before. Briefly, TCGA database: https://portal.gdc.cancer.gov/; UCSC Xena server: http://xenabrowser.net/; GEPIA2: http://gepia2.cancer-pku.cn/; Human Protein Atlas database: http://www.proteinatlas.org/; GDSC database: http://www.cancerrxgene.org/. IMvigor210 (mUC) dataset: http://research-pub.gene.com/IMvigor210CoreBiologies/.

## Code availability

Original code for figures in the paper are available upon request.
