# Supplementary material for: Transcriptional and genetic alterations of cuproptosis-related genes correlated to malignancy and immune-infiltrate of esophageal carcinoma
Source: Cell Death Discov. 2022 Aug 22;8:370. doi: 10.1038/s41420-022-01164-5 (PMC9395517; doi:10.1038/s41420-022-01164-5)
Supplement: Supplementary file 1 — Supplemental Table Legends [file 41420_2022_1164_MOESM1_ESM.docx]

**Supplemental Table Legends**

**Table S1 The list of cuproptosis related genes**

**Table S2 The sequence of siRNA targeting COX7B，SLC25A5 and negative control**

**Table S3 Primers for PCR**
