# Supplementary material for: Transcriptional and genetic alterations of cuproptosis-related genes correlated to malignancy and immune-infiltrate of esophageal carcinoma
Source: Cell Death Discov. 2022 Aug 22;8:370. doi: 10.1038/s41420-022-01164-5 (PMC9395517; doi:10.1038/s41420-022-01164-5)
Supplement: Supplementary file 2 — Gene symbol list [file 41420_2022_1164_MOESM2_ESM.docx]

| **Gene_symbol** |
| --- |
| SLC25A5 |
| CP |
| SLC23A2 |
| NDUFB2 |
| DLD |
| PDHX |
| DLST |
| LIAS |
| ATP7B |
| NDUFA1 |
| COX7B |
| NDUFA2 |
| SLC31A1 |
| FDX1 |
| SLC6A3 |
| LIPT1 |
| DLAT |
| PIH1D2 |
| MITD1 |
| ATP7A |
| CCS |
| LIPT2 |
| ATOX1 |
| NDUFB1 |
| SLC22A5 |

**Table S1 The list of cuproptosis related genes**
