# Supplementary material for: Transcriptional and genetic alterations of cuproptosis-related genes correlated to malignancy and immune-infiltrate of esophageal carcinoma
Source: Cell Death Discov. 2022 Aug 22;8:370. doi: 10.1038/s41420-022-01164-5 (PMC9395517; doi:10.1038/s41420-022-01164-5)
Supplement: Supplementary file 3 — The sequence of siRNA [file 41420_2022_1164_MOESM3_ESM.docx]

**Table S2 The sequence of siRNA targeting COX7B，SLC25A5 and negative control**

| Target | Sense sequence | Antisense sequence |
| --- | --- | --- |
| si-COX7B | 5’-GUCUGUUCCUAGUUAUCUACU-3’ | 5’-UAGAUAACUAGGAACAGACUG-3’ |
| si-SLC25A5 | 5’-GGCAUGUUGUAUUAUAUAACA-3’ | 5’-UUAUAUAAUACAACAUGCCUG-3’ |
| si-Con | 5'-UUCUCCGAACGUGUCACGUTT-3′ | 5'-ACGUGACACGUUCGGAGAATT-3′ |
