# Supplementary material for: Transcriptional and genetic alterations of cuproptosis-related genes correlated to malignancy and immune-infiltrate of esophageal carcinoma
Source: Cell Death Discov. 2022 Aug 22;8:370. doi: 10.1038/s41420-022-01164-5 (PMC9395517; doi:10.1038/s41420-022-01164-5)
Supplement: Supplementary file 4 — Primers for PCR [file 41420_2022_1164_MOESM4_ESM.docx]

| Target | Forward primer | Reverse primer |
| --- | --- | --- |
| RT-qPCR for cell lines | | |
| *GAPDH* | GGAGCGAGATCCCTCCAAAAT | GGCTGTTGTCATACTTCTCATGG |
| *COX7B* | CTTGGTCAAAAGCGCACTAAATC | AAAATCAGGTGTACGTTTCTGGT |
| *SLC25A5* | TTATAGACTGCGTGGTCCGTA | GGCGAAGTTAAGAGCCTGGG |

**Table S3 Primers for PCR**
